# Systematic Identification of Balanced Transposition Polymorphisms in *Saccharomyces cerevisiae*

**DOI:** 10.1371/journal.pgen.1000502

**Published:** 2009-06-05

**Authors:** Dina A. Faddah, Eric W. Ganko, Caroline McCoach, Joseph K. Pickrell, Sean E. Hanlon, Frederick G. Mann, Joanna O. Mieczkowska, Corbin D. Jones, Jason D. Lieb, Todd J. Vision

**Affiliations:** 1 Department of Biology, University of North Carolina at Chapel Hill, Chapel Hill, North Carolina, United States of America; 2 Carolina Center for Genome Sciences, University of North Carolina at Chapel Hill, Chapel Hill, North Carolina, United States of America; 3 Department of Biochemistry, Stanford University, Stanford, California, United States of America; Yale University, United States of America

## Abstract

High-throughput techniques for detecting DNA polymorphisms generally do not identify changes in which the genomic position of a sequence, but not its copy number, varies among individuals. To explore such balanced structural polymorphisms, we used array-based Comparative Genomic Hybridization (aCGH) to conduct a genome-wide screen for single-copy genomic segments that occupy different genomic positions in the standard laboratory strain of *Saccharomyces cerevisiae* (S90) and a polymorphic wild isolate (Y101) through analysis of six tetrads from a cross of these two strains. Paired-end high-throughput sequencing of Y101 validated four of the predicted rearrangements. The transposed segments contained one to four annotated genes each, yet crosses between S90 and Y101 yielded mostly viable tetrads. The longest segment comprised 13.5 kb near the telomere of chromosome XV in the S288C reference strain and Southern blotting confirmed its predicted location on chromosome IX in Y101. Interestingly, inter-locus crossover events between copies of this segment occurred at a detectable rate. The presence of low-copy repetitive sequences at the junctions of this segment suggests that it may have arisen through ectopic recombination. Our methodology and findings provide a starting point for exploring the origins, phenotypic consequences, and evolutionary fate of this largely unexplored form of genomic polymorphism.

## Author Summary

Balanced structural polymorphisms are differences in the relative arrangement of genomic features within species that do not affect DNA copy number. Little is known about their prevalence or importance because they are difficult to observe. Here, we present a novel methodology for systematically identifying such polymorphisms based on the idea that single-copy DNA that occupies different genomic locations in two parents will segregate independently during meiosis and will therefore reveal itself as a copy number difference among a fraction of progeny. Comparative hybridization reveals multiple balanced structural polymorphisms that involve changes to gene order in two strains of yeast; the results are independently validated using paired-end whole genome shotgun sequencing. The longest transposed segment we identify comprises 13.5 kb near the telomere of chromosome XV in the S288C reference strain and contains several annotated genes. We map the location of this polymorphism in the non-reference strain using genome-wide genotypic data, which also reveals an appreciable frequency of ectopic recombination among transposed segment pairs. The breakpoints of the remaining polymorphisms are localized by the paired-end sequence data. Our work provides proof-of-principle for a very general approach to systematically identify all balanced genomic polymorphisms in two different genotypes and is a starting point for understanding the frequency, evolutionary origins, and functional consequences of this seldom-studied class of genomic structural variation in eukaryotes.

## Introduction

Structural rearrangements of the genome are generally defined to include insertions, deletions, inversions, copy number variants, translocations and transpositions greater than one kilobase [1]. While there is substantial interest in the functional and evolutionary role of structural rearrangements [2]–[5] certain classes of rearrangement have been historically difficult to study. In particular, rearrangements that are larger than a standard sequencing read (∼600 bp), yet smaller than can be detected cytologically by microscopy, have been very difficult to detect until recently [6]–[8]. Even now, most studies of genome structural polymorphism focus on unbalanced polymorphisms such as copy number variants, for the simple reason that they are straightforward to detect [9]–[12]. As a consequence, little is currently known about the frequency, mutational mechanisms, phenotypic consequences and evolutionary dynamics of balanced structural polymorphisms in any system, with the exception of those associated with transposable elements [13]–[15].

There is reason to suspect that structural variants might be an important source of natural variation. Altered gene expression with phenotypic consequences may arise through position effects [16] or rearrangements within regulatory regions [3],[5],[17]. Structural variation can also interfere with normal recombination by suppressing crossovers in structural heterozygotes or, more dramatically, by generating recurrent genomic lesions; the latter have been associated with a variety of disease phenotypes in humans [18]. Structural variation may also contribute to postzygotic isolation between incipient species through the production of genetically deficient hybrids [19]. Two striking examples of this have been reported recently. In crosses between *Drosophila melanogaster* and *D. simulans*, sterility in a fraction of hybrid males is caused by the absence of a gene, *JYAlpha,* that is present in both parental strains but located on the fourth chromosome of *D. melanogaster* and the third chromosome of *D. simulans* [20]. In *Arabidopsis thaliana*, recessive embryonic lethality has been observed in an intraspecific cross where the functional copy of an essential gene for histidine biosynthesis is located on different chromosomes in the two parents [21].

Structural rearrangements that affect gene order between closely related species are well known [20], [22]–[24]. Though there are some intriguing individual cases [*e.g.* 25], it is unclear whether such changes are generally adaptive or the consequence of the fixation of neutral and mildly deleterious mutations. One way to address this question would be to study the population genetics of a large and unbiased sample of naturally occurring gene order polymorphisms [26]–[28]. Such an experiment is currently a challenge, however, due to the lack of systematic methods for identifying such polymorphisms genome-wide.

**Figure 1:**
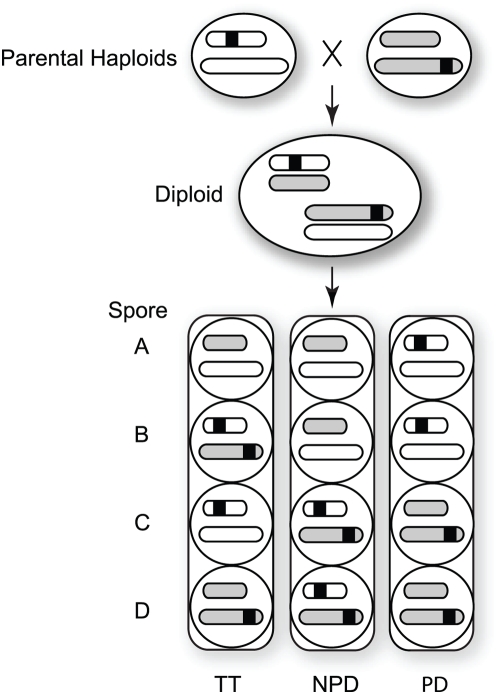
Segregation in a cross between strains carrying a transposed segment. In this example, two haploid parental strains harbor a particular genomic region (black) on different chromosomes. Crossing the parents leads to a diploid that contains two copies of the transposed segment. Sporulation leads to either a tetratype (TT) pattern (one duplication and one deletion); a non-parental ditype (NPD) pattern (two duplications and two deletions), or a parental ditype (PD) pattern (neither duplications nor deletions). The relative frequencies depend on the position of the transposed segment relative to the centromeres in each parent.

Here, we demonstrate an experimental methodology for identifying balanced structural polymorphisms genome-wide at kilobase resolution. It is based on the observation that if a given segment of DNA resides on different chromosomes in a diploid, random assortment of chromosomes during meiosis will cause that segment to appear as a duplication or deletion among a fraction of the resulting haploids (Figure 1). Thus, one can identify such unlinked transposed segments (TS) by using array-based Comparative Genomic Hybridization (aCGH) [6] to measure the copy number of small genomic intervals in the two haploid parents of a diploid, and in the haploid products of diploid meiosis.

The ability to propagate *Saccharomyces cerevisiae* (hereafter “yeast”) strains as haploids allowed us to easily apply this strategy to identify transposed segments genome-wide. We analyzed a cross between polymorphic yeast strains S90 and Y101. The small, well-annotated yeast genome allowed us to characterize the effects of structural variation with greater certainty than would be possible in more complex eukaryotic genomes. The two strains are phenotypically similar in culture and are sexually compatible, as evidenced by F1 tetrads with four viable spores. S90 is nearly identical in gene order and sequence to the sequenced reference strain S288C, while Y101 reportedly lacks ten genes present in S288C [29], and differs from S288C by ∼0.5% at the nucleotide level [30].

## Results

### Identification of Copy Number Polymorphisms between Parental Strains

To identify transposed segments with confidence, we first excluded regions of the genome that differed in copy number or hybridization efficiency between the parental strains. Since a number of deletions in Y101 relative to S288C have been identified previously, these parental hybridizations were also used to assess our technical accuracy in identifying deletions. Genomic DNA from strains S90 and Y101 were separately hybridized to DNA microarrays. In both cases, genomic DNA from strain S288C was used as a hybridization reference. S288C is the original sequenced strain, and the strain from which the microarray probes were derived. The microarrays used in this study represent all coding and non-coding regions in the reference genome with an average probe size of ∼750 nucleotides [31]. We were able to identify all ten of the deletions identified previously in Y101 [29], along with additional putative duplications and deletions (Tables 1 and S1). Some of these apparent deletions may reflect sequence polymorphisms relative to the probe sequence [32],[33].

**Table 1:** The number of microarray probes in each TS confidence class.

Class a	aCGH pattern	No. probes	Percentage
n/a	***Parents*** **:** At least 1 parent not a 1:1 ratio	561	4.7%
n/a	***Parents*** **:** Both 1:1 ratio	8491	70.8%
	***Tetrads*** **:** All 1:1 ratios		
1	***Parents*** **:** Both 1:1 ratios	2390	19.9%
	***Tetrads*** **:** At least 1 duplication or deletion in at least 1 tetrad		
2	***Parents:*** Both 1:1 ratios	529	4.4%
	***Tetrads:*** At least 1 duplication and at least 1 deletion, but in different tetrads		
3	***Parents:*** Both 1:1 ratios	23	0.2%
	***Tetrads:*** At least 1 tetrad with 1 or 2 duplications and 1 or 2 deletions		
		11994	100%

a In order of increasing confidence.

### Identification of Transposed Segments between Parental Strains

Having identified, and eliminated from further consideration, regions of putative copy number variation between the parents, we aimed to identify putative transposed segments. We comparatively hybridized DNA from each of the four spores of six different tetrads against S288C, and identified putative transposed segments as those genomic regions with copy-number differences among the spores (Figure 1). We would expect that, some fraction of the time, a transposed segment would result in a non-parental ditype (NPD) segregation pattern, in which two spores harbor a duplication and two spores harbor a deletion, or a tetratype (TT) pattern, in which one spore harbors a duplication, one spore harbors a deletion, and two spores harbor one copy each. The remaining tetrads would show the parental ditype (PD) pattern, in which each spore harbors one copy. The expected frequency of NPD and TT tetrads cannot be predicted in advance, since the expected frequency is a function of whether the loci are linked in the parental strain and the distance of each locus from the centromere.

**Figure 2:**
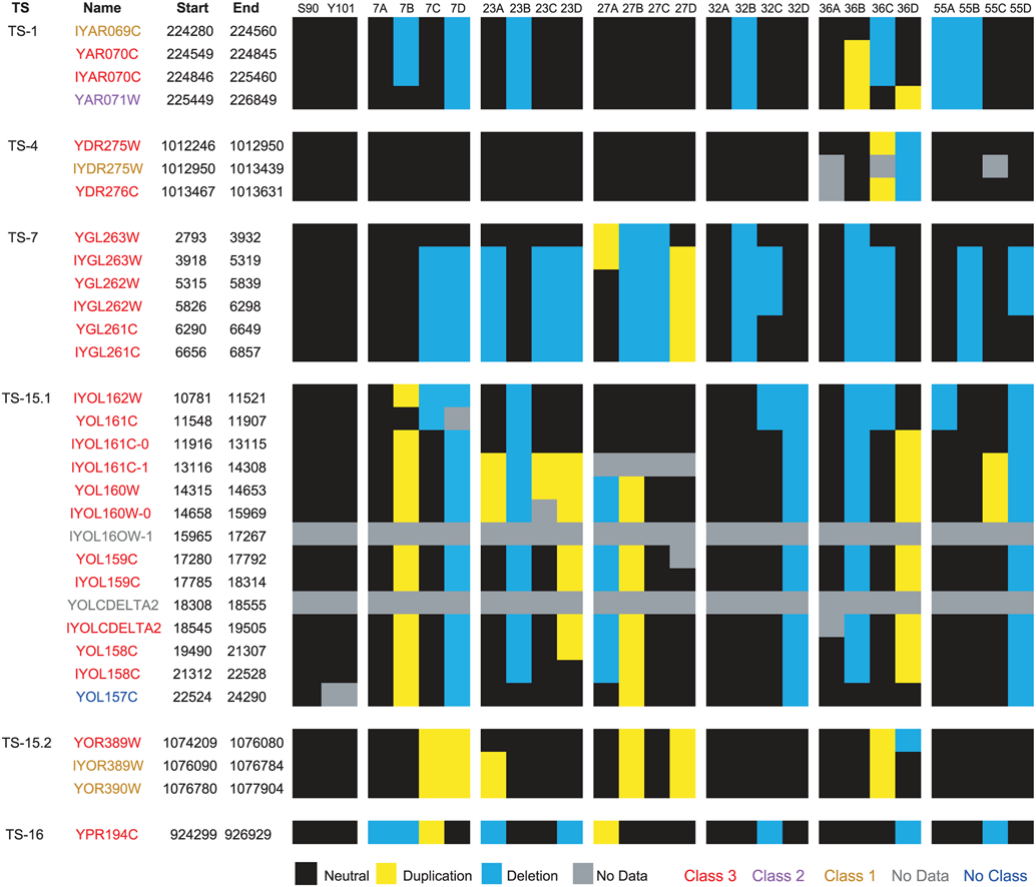
Segregation of the six Class 3 putative transposed segments. Segregation is shown in the two parents (S90 and Y101) and six tetrads (7, 23, 27, 32, 36, and 55) based on aCGH results. The segments reside on S288C chromosomes I, IV, VII, XV, and XVI. Colors represent duplications (yellow), deletions (blue), single-copy (black) and missing data (gray). Probe names are color-coded according to confidence class: 1 (brown), 2 (purple), 3 (red), or not classified (blue).

The measurement noise inherent in DNA microarray hybridizations prevented us from relying entirely on the presence of perfect NPD and TT tetrads to identify transposed segments. Therefore, we initially used a more relaxed criterion to identify genomic regions of potential interest. If any duplications or deletions of that region were observed among the spores, it was placed into one of three classes based on the degree of fit to the expected segregation pattern (Table 1). In Class 1, a single duplication or deletion was observed in one of the tetrads; this class was the most liberal and included approximately 20% of the genome. In Class 2, at least one duplication and at least one deletion were observed, but in independent tetrads; this class included approximately 4% of the genome. In Class 3, the highest confidence category, at least one individual tetrad contained one or two duplications and one or two deletions; this class included 23 regions that covered 0.2% of the genome (Figure 2).

### Characterization of Putative Transposed Segments

**Figure 3:**
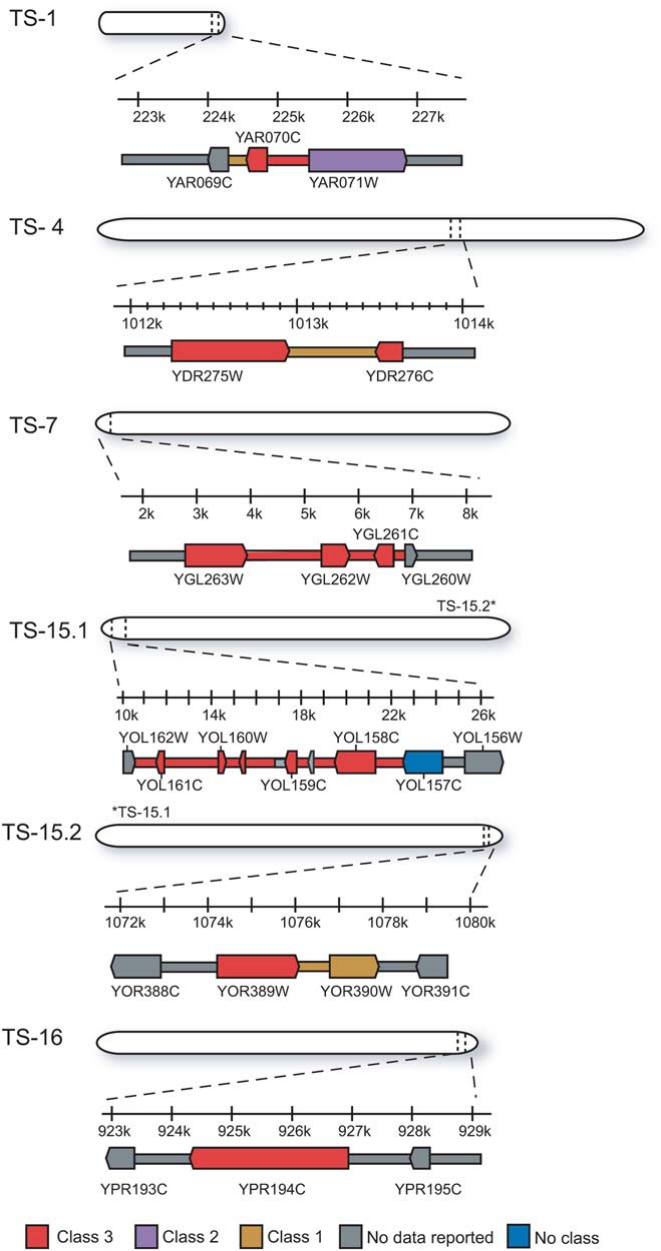
Genomic location and structure of the six Class 3 putative transposed segments. Coordinates are relative to the S288C reference genome. Annotated genes are shown as boxed arrows. Probes are color-coded according to confidence class as in Figure 2.

While some of the Class 1 and Class 2 regions may be true transposed segments, we initially focused our attention on the higher-confidence Class 3 regions. Many of the Class 3 regions were adjacent or nearly so, suggesting that they were part of larger transposed segments. Thus, we grouped Class 3 regions that were located within five kilobases of each other, and that showed compatible segregation patterns, into six distinct putative transposed segments (Figure 2). Each transposed segment (TS) was named based on its chromosomal location in S288C, two on chromosome XV (TS15.1 and TS15.2) and one each on chromosomes I, IV, VII, and XVI (TS1, TS4, TS7, and TS16; Figure 3). They ranged in size from about 1.4 kb to 13.5 kb. Five of the six transposed segments were between 3 and 21 kb from a telomere and demonstrated the Class 3 pattern in at least four out of six tetrads. The lone exception, TS4, was also the smallest putative transposed segment and exhibited the Class 3 pattern in just one tetrad. Collectively, the six transposed segments contained a total of 15 annotated genes (seven ‘verified’, six ‘uncharacterized’, and two ‘dubious’) and one transposable element (Table 2).

**Table 2:** Genes within Class 3 putative transposed segments, based on annotations in Saccharomyces Genome Database (SGD) November 2008 [67].

TS	Gene Name	Synonym	Feature Type	Description	GO Molecular Function	GO Biological Process
1	YAR070C		ORF, Dubious	Dubious open reading frame unlikely to encode a protein, based on available experimental and comparative sequence data	unknown	unknown
1	YAR071W	PHO11	ORF, Verified	Phosphate metabolism	acid phosphatase activity	phosphate metabolic process
4	YDR275W	BSC2	ORF, Verified	Bypass of stop codon	unknown	unknown
4	YDR276C	PMP3	ORF, Verified	Plasma membrane proteolipid	unknown	cation transport/ regulation of membrane potential
7	YGL263W	COS12	ORF, Verified	Conserved Sequence	unknown	unknown
7	YGL262W		ORF, Uncharacterized	Putative protein of unknown function; not an essential gene	unknown	unknown
7	YGL261C	PAU11	ORF, Uncharacterized	Putative protein of unknown function; mRNA expression appears to be regulated by SUT1 and UPC2; seriPAUperin	unknown	unknown
15.1	YOL161C	PAU20	ORF, Uncharacterized	Hypothetical protein; seriPAUperin	unknown	unknown
15.1	YOL160W		ORF, Dubious	Dubious open reading frame unlikely to encode a protein, based on available experimental and comparative sequence data	unknown	unknown
15.1	YOL159C		ORF, Verified	Soluble protein of unknown function; deletion mutants are viable and have elevated levels of Ty1 retrotransposition and Ty1 cDNA	unknown	unknown
15.1	YOLCDELTA2		long terminal repeat	Ty1 LTR	not available	not available
15.1	YOL158C	ENB1	ORF, Verified	Enterobactin	ferric-enterobactin transmembrane transporter activity	ferric-enterobactin transport
15.1	YOL157C		ORF, Uncharacterized	Putative protein of unknown function	unknown	unknown
15.2	YOR389W		ORF, Uncharacterized	Putative protein of unknown function; expression regulated by copper levels	unknown	unknown
15.2	YOR390W		ORF, Uncharacterized	Putative protein of unknown function	unknown	unknown
16	YPR194C	OPT2	ORF, Verified	Oligopeptide transporter; member of the OPT family, with potential orthologs in *S. pombe* and *C. albicans*	oligopeptide transporter activity	oligopeptide transport

### Characterization of TS15.1

**Figure 4:**
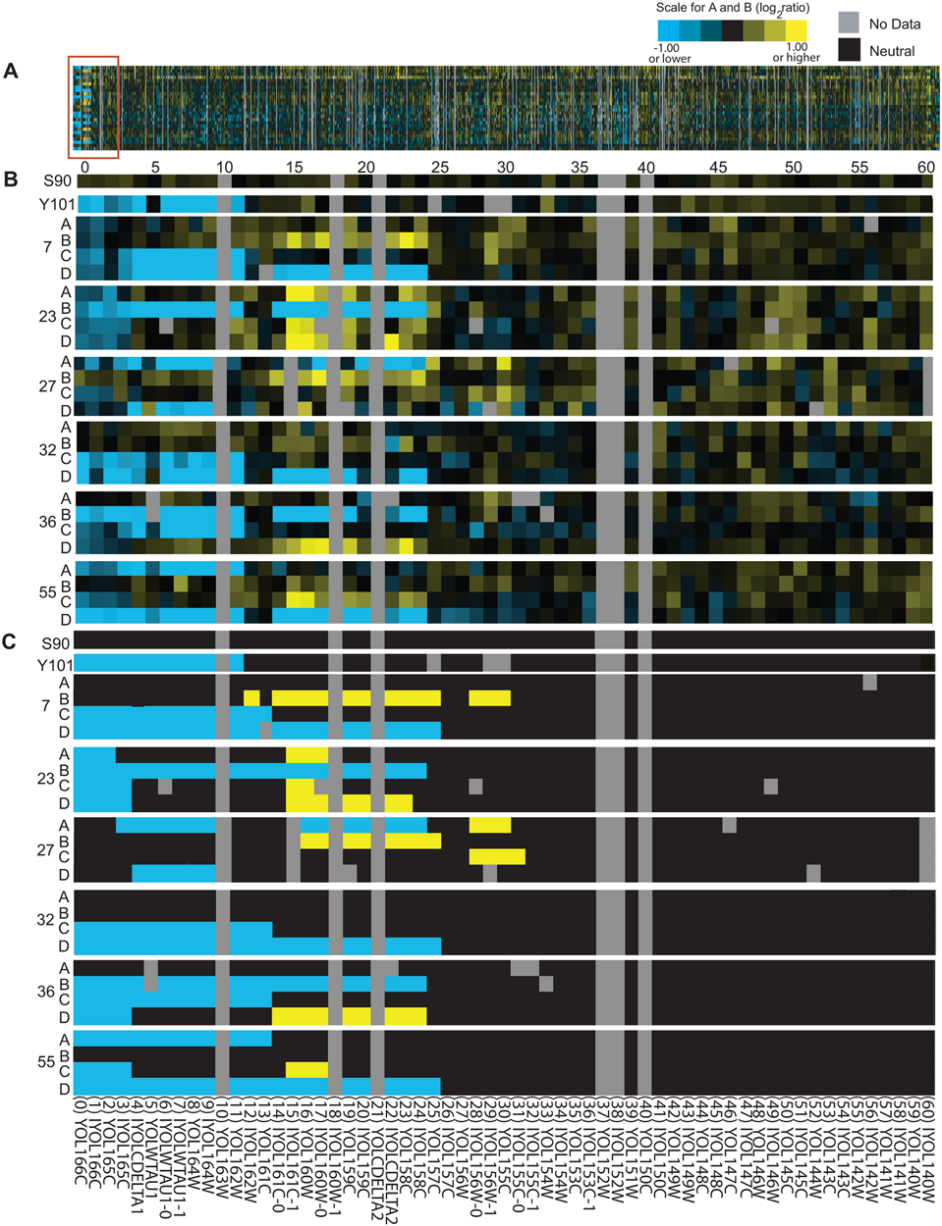
Detailed characterization of the TS15.1 region. (A) aCGH data for all tetrads across chromosome XV. The area within the red box contains TS15.1, and is shown in more detail in panel B, (B) Raw aCGH data and (C) inferred duplications (yellow) and deletions (blue).

We sought to determine the endpoints of TS15.1, the largest of the six segments, more precisely by manual inspection of the hybridization data (Figure 4). The segment was initially identified by eleven closely linked Class 3 regions, including 12–17, 19, 20, and 22–24. Probes 18 and 21 had been excluded from consideration initially because they were not present on the particular batch of microarrays we used.

The positions of the TS15.1 endpoints were ambiguous on the basis of the aCGH data alone. While the most distal duplication (relative to the centromere) was at probe 12, deletions continued all the way to the telomere in Y101 (probes 0–11), in addition to several of the spores. At the proximal end of TS15.1, probe 25 had been excluded due to missing data from Y101 and probes 26 and 27 showed no evidence for copy number variation. While probes 28–31 had duplications in three of the spores, only one of these spores was duplicated in the core regions of TS15.1.

This ambiguity motivated us to further characterize the endpoints of TS15.1 by a PCR assay. We tested for amplification of an appropriately sized product from the parents and the spores using primers that corresponded to the ends of the microarray probes. While detection of duplications requires a more quantitative assay, our methodology could easily identify deletions. Amplification within a transposed segment should fail in a spore harboring a deletion while succeeding in both parents. Amplification could also fail if the primers span a transposed segment endpoint or if the primer sites have diverged between the two strains, but these cases can be distinguished from true deletions by a lack of amplification in Y101, since the primers are designed to match the S288C reference sequence.

**Figure 5:**
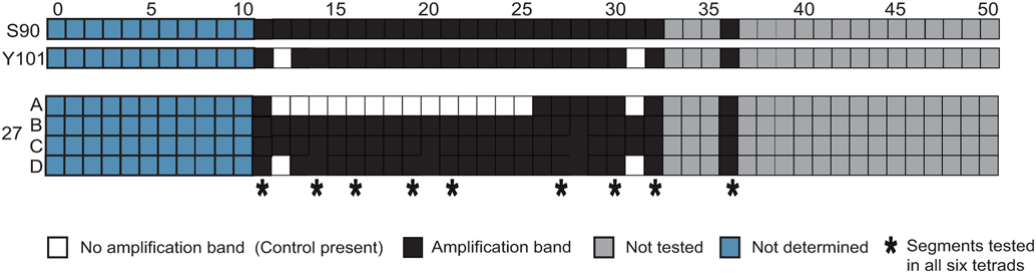
Results of PCR assays for the boundaries of TS15.1 in tetrad 27. Based on the aCGH data, deletion of TS15.1 was expected in spore 27A. Probes tested in all six tetrads are indicated by asterisks. Due to the repetitive nature of subtelomeric segments 0–10, PCR assays produced ambiguous results (marked as “not determined”). See also Table S3.

**Figure 6:**
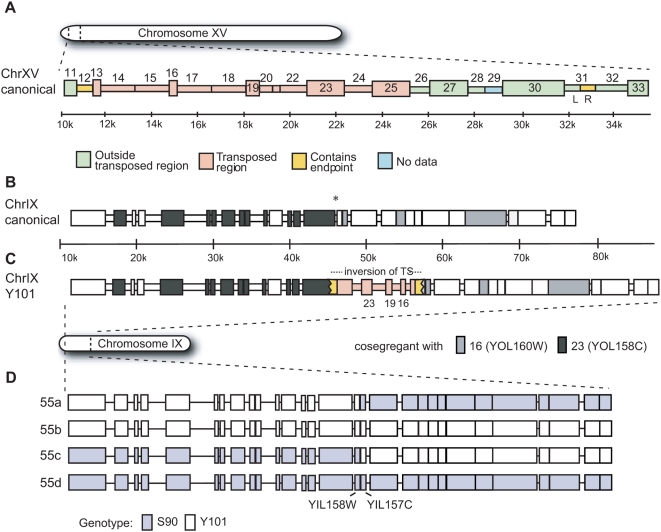
Model for the position and structure of TS15.1 in the parental strains. Boxes represent annotated genes (tall) and intergenic regions (short). Systematic gene names for probe numbers are given in Figure 4. (A) Chromosome XV of S288C. Probes are color-coded as being either outside the transposed region (green), within the transposed region (pink), or as containing an endpoint (yellow). (B) Chromosome IX of S288C. The TS breakpoint is indicated by an asterisk. (C) The inferred position and orientation of the TS is shown for Y101 using the color scheme from panel A. The shading in panels B and C shows the cosegregation pattern of Y101 chromosome IX probes with those on S288C chromosome XV according to the GMS data. (D) Genotyping results for the chromosome IX segment in tetrad 55, showing evidence of recombination between YIL158W and YIL157C.

Initially, we tested primer pairs corresponding to probes 11 through 32 and probe 36 in the two parental strains, the reference strain, and the four spores from tetrad 27 (Figure 5, Tables S2 and S3). Amplification was obtained from all genotypes using primer pairs from probes 11, 26–30, 32, and 36. Primers corresponding to probes 13 through 25 failed to amplify products only in spore 27A, consistent with the hypothesis that this spore did not inherit TS15.1 from either parent. Probes 12 and 31 failed to amplify in spore 27A and D, and also in Y101, indicating that segments 12 and 31 are candidate endpoints for TS15.1. To map the right endpoint more finely, we designed new primers to split probe 31 into two halves, 31L and 31R. The results support 31L as being external to the transposed segment, and identify 31R as containing the endpoint (as illustrated in Figure 6).

The pattern of amplification in probes 12 and 31R is consistent with the endpoints of the transposed segment occurring within these two intervals. However, since probes 26–30 amplified in all genotypes, they appear to be external to the transposed segment. To confirm this surprising result, we used primers corresponding to probes 11, 14, 16, 19, 21, 27, 30, 32, and 36 in the other five tetrads. In all cases, the amplification results were consistent with the aCGH-predicted duplications and deletions in probes 13–25 and the PCR-predicted endpoints in probes 12 and 31R (data not shown). The presence of an apparent endpoint within probe 31R rather than probe 25 suggests that TS15.1 region differs not only in genomic location, but also in structure, between the two strains, possibly through an inversion of ∼6 kb. Thus, we conclude that TS15.1 comprises the segments of the genome covered by probes 13–25 and portions covered by probes 12 and 31R, for a total of about 15 kb of DNA originating approximately 12 kb from the left end of the chromosome XV reference sequence (Figure 6).

### TS15.1 Resides on Chromosome IX in Y101

We can infer the position of a transposed segment in S90 based on its position in the genome assembly of S288C, but its position in Y101 is unknown. To map the transposed segment in Y101, we identified genomic regions that co-segregated with the transposed segment in F1 spores. We obtained parent-of-origin information for 6,215 open reading frame (ORF)-based probes in each spore using a second microarray-based technique, genomic mismatch scanning, or GMS [34]–[36]. With these data, we can also identify meiotic crossover events that may have occurred within the transposed segment. We focused on the two genes within TS15.1, YOL158C (probe 23) and YOL160W (probe 16). In the F1 haploids, alleles of YOL158C derived from Y101 perfectly cosegregated with eleven genes near the left telomere of chromosome IX, while alleles of YOL160W derived from Y101 perfectly cosegregated with three genes immediately adjacent to the same region on chromosome IX (Figure 6).

**Figure 7:**
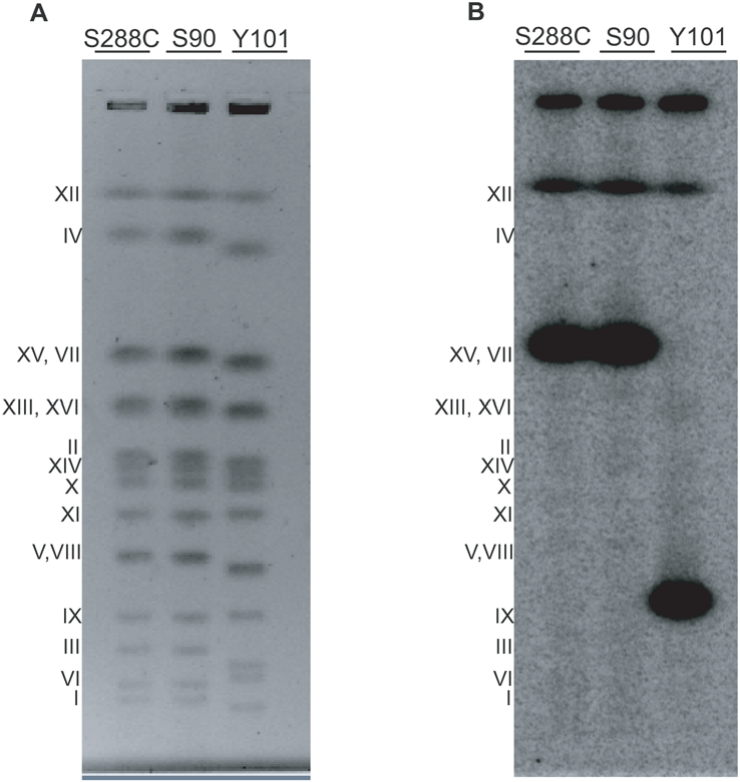
Experimental validation of the transposition of TS15.1 in strain Y101. (A) Ethidium bromide stained chromosomes of S288C, S90, and Y101 separated by pulsed field gel electrophoresis. (B) A Southern blot using YOL158C (from within TS15.1) as hybridization probe. In Y101, the probe hybridizes to chromosome IX but not chromosome XV. Note that 3 pairs of chromosomes cannot be distinguished on the gel, including chromosomes XV and VII.

To verify the predicted location of TS15.1 on chromosome IX in Y101, the chromosomes of both S90 and Y101 were separated using pulsed-field gel electrophoresis (PFGE) and subjected to Southern blotting with a probe amplified from within TS15.1. The probe hybridized to the band that includes both chromosomes XV and VII in S288C and S90, while it hybridized to the chromosome IX band in Y101 (Figure 7). A secondary signal, perhaps the result of cross-hybridization to the rDNA repeats, was observed from chromosome XII in all strains. Thus, the PFGE and GMS-data are both in agreement that TS15.1 is located on chromosome IX in Y101.

Despite the location of TS15.1 on two different chromosomes in the parental strains, and evidence for structural heterogeneity between the two alleles, two meiotic crossovers appear to have occurred very near, possibly even within, the TS. One of the events was observed in tetrad 32 between gene YIL154C and gene YIL155C and one was observed in tetrad 55 between YIL158W and YIL157C (the latter illustrated in Figure 6). The segregation patterns leads us to infer that the orientation of the segment in Y101 is opposite to that in S90, *i.e.* that YOL157C is distal to YOL161C (results not shown).

### Evidence That S288C Represents the Ancestral Organization of TS15.1

The TS15.1^S288C^ arrangement is seen not only in S90 but in the genome assemblies of two other sequenced *S. cerevisiae* strains: the wine strain RM11-1a [37] and YJM789, a strain isolated from the lungs of an AIDS patient with pneumonia [32]. While additional strains have been sequenced by the Saccharomyces Genome Resequencing Project [38],[39], the genome assemblies have used S288C as a template, and thus are not informative regarding structural differences relative to that template. Instead, we examined the sequence reads that spanned the breakpoints, segments 12 and 31R. We found no evidence for the null allele in any of the strains, suggesting that TS15.1^S288C^ is by far the more common arrangement among present-day strains.

**Figure 8:**
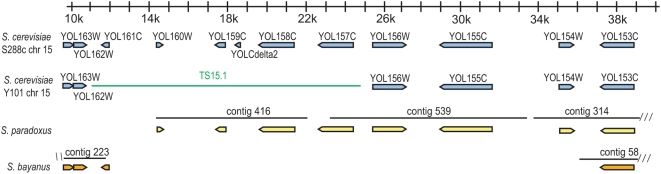
Comparative genomic evidence that S288C harbors the ancestral form of TS15.1. Annotated genes (open boxed arrows) are shown for *S. paradoxus* and *S. bayanus* contigs relative to the known structure of the region in S288C and the inferred structure in Y101. *S. paradoxus* contig 539 matches the gene order of S288C across the proximal endpoint of TS15.1 while *S. bayanus* contig 223 matches the gene order of S288C across the distal endpoint. The green bar shows the position of the gap on chromosome XV of Y101. Each scalebar tick represents 1 kilobase.

To determine the ancestral state for TS15.1, we examined the genome sequence of *S. paradoxus* and *S. bayanus* [24],[40]. In the initial genome assembly, *S. paradoxus* “contig 539” contains homologs to the genes YOL157C (probe 25), YOL156W (probe 27) and YOL155C (probe 30), which span the proximal endpoint of the transposed segment and are arranged in the same order and orientation as in S288C (Figure 8). Likewise, *S. bayanus* contig 223 contains homologs to the genes YOL163W (probe 10), YOL162W (probe 11) and YOL161C (probe 13) which span the distal endpoint of the transposed segment are also arranged in the same order and orientation (Figure 8). While it is not possible to compare the genome organization distal to the transposed segment due to the incompleteness and fragmentation of the assemblies in this region for *S. paradoxus and S. bayanus*, this nonetheless strongly suggests that TS15.1^S288C^ is the ancestral state.

### Cross-Platform Validation Using Paired-End Sequencing

To validate the aCGH results using an independent method, we obtained 28× coverage paired-end Illumina sequencing of Y101. The ends of each mapped genomic fragment were separated by 243±35 (mean±standard deviation) base pairs. To detect transposition events, we looked for instances in which the two ends of a paired-end read mapped to a different chromosome, or cases in which they mapped more than 5 kb apart from each other on the same chromosome, relative to the S288C reference sequence.

We detected 40 such discordant paired-end sequences, each represented by multiple independent sequence reads. The start and end points of these genomic segments are given in Table S4. The end sequences can be used to locate and orient the position of the corresponding segment in Y101, and to examine the location of the breakpoints in both strains at fine resolution. For instance, the location of TS15.1 on chromosome IX in Y101 is confirmed by the paired-end data. Eighteen of the 40 discordant paired-ends map to different chromosomes, and thus may represent transposed segments. Interestingly, the paired-end data do not support the idea, suggested by the Class 3 transposed segments, that such rearrangements occur predominantly within subtelomeric regions. There are 21 regions for which the paired-end sequences occur at two loci on the same chromosome separated by only 5 to 15 kb, indicative of local rearrangements. In only one case are the paired-ends found on the same chromosome at a distance greater than 50 kb. Overall, four of the six Class 3 TS were validated by the paired-end sequencing data: TS7, TS15.1, TS15.2, and TS16. For the remaining two Class 3 predictions (TS1 and TS4), the many independent paired-ends that spanned the junctions mapped to the same locus on the reference genome within the specified tolerance. Therefore, we conclude that TS1 and TS4 are false positives, yielding an overall specificity of 67% for calling TS by their Class 3 status. The paired-end sequencing data provide evidence for rearrangements in ten of the 529 Class 2 array features, yielding a specificity of only 1.9% for these lower-confidence predictions. The number of rearranged features identified by paired-end sequencing data among those categorized as Class 0 or Class 1 is 0.09% (10 rearranged features out of a total of 11,442 Class 0 and Class 1 features).

## Discussion

Until recently, genotyping technology has been unable to measure the prevalence of variation in the genomic location of kilobase-sized single-copy segments. Here, we have shown that it is possible to systematically identify balanced transpositions among members of the same species at kilobase resolution throughout the genome by detection of copy-number changes in the segregating progeny of a genetic cross. Furthermore, we have shown that the location of a balanced transposition can be mapped with genome-wide SNP segregation data from the same cross. By applying this methodology to two divergent strains of *S. cerevisiae*, we have found a modest number of unlinked gene order polymorphisms. The method predicted six transposed segments, of which four were validated by paired-end whole-genome shotgun sequencing. The four validated transposed segments range from less than 3 kb in size to more than 13 kb, and collectively contain nine annotated genes. Furthermore, there is evidence from the paired-end sequencing data for at least 14 other transposed segments segregating in this cross.

Our approach takes advantage of aCGH, a technology already in wide use. Dunham *et al.* [10] previously demonstrated the ability to detect large balanced translocations in experimentally evolved yeast strains through aCGH of novel PFGE-isolated fragments. Similarly, Casaregola *et al.* [41] used PFGE to detect pre-existing rearrangements in a cross between two yeast strains. In contrast, the approach described here can easily be adapted to organisms with larger genomes, for which PFGE is not generally practical. While yeast is a convenient test system due to the fact that copy number can be assayed among the spores within a tetrad, the approach can be applied to diploid organisms such as Drosophila, mouse and maize. Copy number variation among members of a standard experimental mapping population, such as an F2 or recombinant inbred line population, also permits detection of balanced rearrangements, as has been previously recognized [42].

We have also confirmed that paired-end sequencing is a powerful alternative platform for examining genomic and structural rearrangements at a high-resolution, genome-wide level. Paired-end sequencing can be used to detect not only transpositions and translocations, but also inversions within a chromosome [43]. However, the utility of paired-end sequencing is limited to systems for which a reference genome is available, while the use of copy number variation among segregating progeny can, in principle, be applied more widely (*i.e*. by assaying the copy number of sequence tagged sites on a dense genetic map).

The paired-end sequencing data allowed us to reject one-third of our Class 3 predictions, and to detect an additional 14 putative inter-chromosomal rearrangements and 22 intra-chromosomal rearrangements, of which 10 had been categorized as Class 2 due to duplications and deletions occurring in different tetrads within the aCGH data. Interestingly, all the intra-chromosomal rearrangements within 15 kb were found to be associated with transposable element repeat sequences. These highly localized rearrangements would likely have been invisible even to the most noise-free aCGH data, since the affected loci would not have segregated independently.

### Origins of Balanced Structural Variants

All four of the confirmed transposed segments found by aCGH were located near telomeres, which are known to be susceptible to internal rearrangement [44], sister chromatid exchange [45], and interchromosomal exchange [46]. This is consistent with the unexpected observation of illegitimate recombination between the transposed segment on chromosomes IX and XV. Interestingly, the genes concerned (YIL154C and YIL155C) have been previously identified as hotspots for double-strand breaks [47]. In addition, subtelomeric regions are commonly considered to be permissive of structural rearrangements [24]. For instance, Wei *et al.* [32] reported an 18 kb subtelomeric segment on chromosome VI in S288C that is found on chromosome X in YJM789. The relatively few subtelomeric genes are seldom transcribed at a high level and are frequently silenced [48],[49]. None of the genes within the confirmed Class 3 TS are essential for growth in rich medium [50]. Deficiencies arising from the segregation of the structural polymorphisms we identified would therefore not be expected to cause gross phenotypic effects in lab culture. While this is consistent with the viability of all the tetrad products examined in this study, the results are biased by the initial selection of complete tetrads for the original GMS study. Of the 312 tetrads originally assayed from the cross, the proportion found to have 0, 1, 2, 3, and 4 viable spores was 5.4%, 3.2%, 13.1%, 30.8% and 47.4%, respectively, yielding an overall viability of 77.9% (J. McCusker, pers. comm.).

On the other hand, relative to the overall gene density in yeast of 0.5 gene/kb [51], we find higher-than-average density in three of the subtelomeric transposed segments (TS7: 0.71 gene/kb; TS15.2: 1 gene/1.8 kb, TS16: 0.55 gene/kb) and lower-than-average density in only one (TS15.1: 0.37 gene/kb). Thus, the transposed segments do not, as a general rule, occur in gene-poor regions. In fact, many of the TS junctions, as inferred from paired-end data (Table S4), are located within the boundaries of annotated exons. Thus, both a high rate of subtelomeric structural mutation and relatively weak purifying selection may both contribute to the maintenance of polymorphism for the transpositions catalogued here.

There are no obvious shared genomic features among the transposed segments apart from their subtelomeric positions, and even the subtelomeric bias is absent among the putative rearrangements that are observed only in the paired-end sequencing data. There is a fragmented *TY* retrotransposon sequence located inside TS15.1 (YOLCDELTA2), but retrotransposons are not found near any of the other transposed segments despite the ability of such elements to generate genomic rearrangements [13],[52]. Alternatively, duplicate genes and other low-copy repeats can initiate ectopic exchange [53]. The two junctions of TS15.1, as inferred from the paired-end sequencing data (Table S4), show strong sequence similarity. The chromosome XV junction is in the middle of YOL155C (HPF1), and the chromosome IX junction is in the middle of YIL169C, a homolog of HPF1 with high sequence similarity (*E* = 2.6e−172 in a BLASTP search among annotated yeast proteins). Furthermore, both are adjacent to HXT genes that have 97% identity to each other at the nucleotide level. While repetitive gene families are known to be well-represented in subtelomeric regions [48], this is the only pair of junctions among the four confirmed Class 3 TS for which we found evidence of sequence similarity. Thus, non-allelic homologous recombination appears to underlie at least some of the structural mutations observed, but we cannot exclude a role for other mechanisms such as repair of spontaneous double stranded breaks by non-homologous end-joining [54],[55].

### Functional and Evolutionary Consequences of Balanced Transposition Polymorphisms

Further work will be necessary to determine the phenotypic consequences of these structural polymorphisms, and indeed of balanced structural polymorphisms in general. Interruption of functional genes and generation of fusion genes are likely to have phenotypic effects in some cases [54]. There is no direct evidence of such rearrangements within TS15.1, but we do not have adequate data to rule them out in the other transposed segments.

Position effects on expression are also likely [17]. Expression can be altered not only for the transposed genes, but also for neighboring genes [5]. Some effects on expression could be evolutionarily significant even if phenotypically subtle. For example, it has been proposed that selection favors the clustering of essential genes that are sensitive to stochasticity in expression levels [56]. Naturally occurring transposed segments provide an opportunity to study such context-sensitivity of expression for a large number of genes in a variety of genetic backgrounds. Other classes of phenotypic effects may be ephemeral on the population level, but nonetheless dramatic for affected individuals. Non-allelic homologous recombination between transposed segments at different loci can lead to recurrent genetic lesions [57], and segregation of transposed segments in the population can lead to recurrent genetic duplications and deficiencies such as those observed among the tetrads studied here.

If these phenomena affect organismal fitness, they will influence the frequency of balanced polymorphisms in natural populations. We would expect most transposed segments to be held at low frequencies by purifying selection (due to the deleterious fitness effects of ectopic recombination and dosage imbalance) or have dynamics that are governed largely by genetic drift. At the same time, it is likely that some small fraction of transposed segments are adaptive, as has been suggested for the compound structural rearrangement leading to the cluster of genes involved in allantoin degradation pathway in *S. cerevisiae* and *S. castellii* [25]. Since universally deleterious and adaptive rearrangements are unlikely to remain polymorphic for an extended period of time, balanced transpositions that are maintained at intermediate frequencies must either have a high recurrent mutation rate (to counteract genetic drift) or be subject to conditional fitness effects. The latter class of polymorphisms, typified by the classical inversion polymorphisms in *Drosophila pseudoobscura* [58], would be particularly interesting to uncover. More recently, Feuk *et al.* [59], reported that 13% of the inversions that they detected in a comparison of the human and chimp genome, with lengths between 1 Kb and 1 Mb, were segregating at appreciable frequency (minor allele ≥5%) within humans, though it is not yet clear if this small subset of inversions are neutral or deleterious polymorphisms still in transit, or actually being maintained by balancing selection.

The derived TS15.1 chromosome has currently been observed in only one strain, although Schacherer *et al.* [60], in a survey of diverse strains of yeast, report deletions of the genes within TS15.1 in as many as 19 (30%) of the 63 strains surveyed (Table 3). They also report that genes within TS7 and TS15.2 may also be deleted with appreciable frequency. It is not yet clear whether the high frequency of deletions observed at these loci reflects segregation of the same alleles observed here, or whether these instead are derived from some number of independent rearrangement events. In the former case, it would be interesting to explore whether any of these three TS are being maintained by selection, while in the latter case, it would suggest the equally interesting conclusion that the frequency dynamics of these gene deletions are being driven by recurrent mutation.

**Table 3:** Deletions in putative transposed segments observed among 63 *S. cerevisiae* strains by Schacherer *et al*. [60].

Transposed segment	Gene name	No. of strains in which deletion is observed
TS1^a^	YAR071W	2
TS7	YGL263W	10
TS7	YGL262W	11
TS15.1	YOL160W	18
TS15.1	YOL159C	19
TS15.1	YOL158C	2
TS15.2	YOR389W	39
TS16	YPR194C	2

a not validated

In conclusion, we have developed a systematic method to identify gene order differences between two divergent yeast strains. Our results show that the genomes of two divergent strains of *S. cerevisiae* are largely collinear, but do harbor a modest number of gene-containing subtelomeric transpositions that are several kilobases in size. These findings raise important questions about the phenotypic consequences and evolutionary dynamics of balanced structural polymorphisms, not only in yeast but also in other natural populations.

## Materials and Methods

### Yeast Strains

We examined six tetrads from a cross between two *S. cerevisiae* strains. One strain is YJM826/S90m, a spontaneous Gal+ derivative of S1, which is isogenic with the S288C genome reference strain. The other strain is Y101 (hoΔ MATα gal3 Mal Suc Bio), a haploid derivative of YJM627/Y55 (HO gal3) said to have been isolated from a French vineyard in the 1930s by Oyind Winge [29],[30]. Cultures of the parental strains and tetrads were kindly provided by P. O. Brown.

### Genomic DNA Purification

For aCGH, twenty-milliliter cultures of each sample were grown in YPD medium (yeast extract 10 g/L, peptone 20 g/L, and 2% dextrose) to OD_600_ greater than one. Cells were collected and resuspended in TE (700 µL of 200 mM Tris, 50 mM EDTA, pH 8) and frozen overnight. To digest cell walls, the cells were resuspended in 535 µL of 20 mg/ml Zymolyase, 1.2 M Sorbitol, 20 mM HEPES (pH 7.5) and incubated for 60 minutes at 37°C. Collected cells were resuspended in TE+10 mg/ml RNaseA at 65°C for 30 minutes. 200 µL 5 M potassium acetate was added and cells were incubated on ice for 1 hour. The lysed cells were then centrifuged at 14K rpm for 10 minutes to pellet debris. Genomic DNA was ethanol precipitated from the supernatant and resuspended in 200 µL TE (pH 8). The solution was then sonicated to fragment DNA to an average size of ∼500 bp. DNA was purified using a YeaStar Genomic DNA Kit (Zymo Research, cat # D2002) and concentrations were determined by absorption spectroscopy with PicoGreen dye (Invitrogen). DNA templates suitable for PCR were obtained as previously described [61]. For paired-end sequencing, five ml of YPD was inoculated with a single colony, incubated overnight at in 30°C, and centrifuged. The pellet was resuspended in 500 µl of lysis buffer (1.2 M sorbitol in 0.1 M KPO_4_, pH 7.4), 30 µl Zymolyase (20 mg/ml), incubated at 37°C for 45 minutes and centrifuged again. The pellet was resuspended in 500 µl of 50 mM Tris+10 mM EDTA, 1% SDS with 3 µg of RNase and incubated at 65°C for 25 min. 200 µl of 5 M potassium acetate was added, then the solution was incubated on ice for 40 min. The pellet was washed with isopropanol and 70% ethanol and left overnight at room temperature for drying. Libraries were prepared for paired end sequencing on an Illumina GA2 sequencer using standard Illumina protocols (Illumina, San Diego, CA).

### DNA Labeling for aCGH

Twenty microliters of 2.5× random primer/reaction buffer mix (125 mM Tris 6.8, 12.5 mM MgCl_2_, 25 mM 2-mercaptoethanol, 750 mg/mL random octamers) were added to 21 µL of genomic DNA. The DNA was incubated at 100°C for 5 minutes and then placed on ice for 10 minutes. Five microliters of 10× DNTP mix (1.2 mM dATP, dGTP, dCTP, 0.6 mM dTTP, 10 mM Tris 8.0, 1.0 mM EDTA) were added to the genomic DNA. Test samples were labeled by addition of 3 µL 25 nmol of Cy5-dUTP (Amersham) to the reaction mix, and the reference sample, S288C, was labeled by addition of 3 µL 25 nmol of Cy3-dUTP (Amersham) to the reaction mix. One microliter 40 U/µL Klenow was added to each sample. The DNA was incubated at 37°C for two hours and the labeling reaction was stopped with 5 µL 0.5 M EDTA pH 8.0. For each microarray, we purified 5 µg of labeled DNA using a DNA Clean and Concentrator System-5 (Zymo Research, cat #D4004).

### DNA Microarray Design and Hybridization

The yeast tiling microarrays were identical to those previously described [31],[62]. Briefly, each gene probe extended from start codon to stop codon. Probes for intergenic regions and other features (including rDNA, tRNA, transposons, transposon long terminal repeats, introns, telomeres, and centromeres) conformed to the boundaries as annotated by the *Saccharomyces* Genome Database (SGD) in 2000. The PCR products were printed on poly-L-lysine coated glass slides by a robotic arrayer as described [63].

DNA was hybridized to the microarrays as previously described [63]. In all cases, S288C was used as the reference for competitive hybridization. Images were acquired using a GenePix 4000B scanner and software (Axon Instruments). Raw and processed aCGH data were loaded into the University of North Carolina Microarray Database (http://genome.unc.edu). The hybridization signal of each probe was recorded as the log_2_ normalized ratio of the median pixel intensity for the sample relative to the reference. Only probes composed of pixels with consistent ratio values (regression *r* ^2^>0.6) and with gel-verified PCR products were used for analysis.

### aCGH Data Analysis

The vector of values from each hybridization was normalized such that the median ratio of all probes relative to the S288C reference was zero. Duplications and deletions in the aCGH data were then detected in a stepwise manner using ChIPOTle version 1.0 [64], using a 1-kb window and a 250 bp step size. First, deletions were identified by using the reciprocal of the ratios (reference/sample). The deletions were then removed from the dataset, and the dataset was re-normalized such that the median ratio of all remaining probes was zero. ChIPOTle was then run on the re-normalized data to detect duplications. Segments for which *p*<0.001 after Bonferroni correction were classified as deletions or duplications, accordingly. A custom Perl script (available upon request) was used to identify potential transposed segments by classifying each ChIPOTle window according to the pattern of duplications and deletions observed in the parents and tetrads, as described in Results. Heatmap visualizations were generated using Java Treeview [65].

### PCR Assays

The initial primers for the PCR assay were the same as those used to amplify the probes for the microarray, with the exception of probes 14, 19, 22, 30, 31, and 32 (Table S2). For probes 31L and 31R, primer pairs [31 forward and internal] and [31 internal and reverse] were used, respectively. Twenty-five µL PCR reactions were prepared at final concentrations of 1× Mg free buffer (Promega), 0.1 unit/µL Taq DNA polymerase (Promega), 0.25 mM dNTP, 2 mM MgCl2 (Promega), 0.14ng/µL genomic DNA, 20 pmol amplification primers (IDT), and 20 pmol positive control primers (IDT). The reactions were carried out with an initial incubation at 92° for one minute, followed by 36 cycles of 92°C for 30 sec, 56°C for 45 sec, and 72°C for 3.5 min.

### Genome Mismatch Scanning

GMS is a procedure for biochemical enrichment of genomic regions in which individuals share identical alleles, *e.g.* two parents and their progeny. GMS data for the six tetrads was obtained during the course of a companion experiment (C. McCoach, K. Hayashibara, X. Cui, S. Elashoff, E. Ray, J. McCusker, J. DeRisi, D. Siegmund and P. Brown, unpubl.). The procedure was performed as described [35]. In brief, heterohybrid DNA molecules formed between genomic DNA fragments from two individuals were purified by differential methylation and endonuclease restriction. Heterohybrids containing mismatches were then removed by the *E. coli* mismatch repair enzymes Mut H, Mut L, and Mut S. This was done using heterohybrid DNA created from each of the 24 spore strains and the two parents (a total of 48 samples). The hybridization pools for each spore were differentially labeled and comparatively hybridized against each parent to a microarray containing probes for 6,145 genes. The probability of inheritance of each gene from either parent was calculated from the GMS data using a hidden Markov model approach to be described elsewhere (C. McCoach, K. Hayashibara, X. Cui, S. Elashoff, E. Ray, J. McCusker, J. DeRisi, D. Siegmund and P. Brown, unpubl.).

Let *X* ^S90,P^ and *X* ^Y101,A^ denote the S90 (present) and Y101 (absent) alleles, respectively, for a given transposed segment at position *X;* and let *Y* ^S90,A^ and *Y* ^Y101,P^ denote the S90 (absent) and Y101 (present) alleles, respectively, at position *Y*. The transposed segment will be duplicated in spores with genotype *X* ^S90,P^/*Y* ^Y101,P^, deleted in spores with genotype *X* ^Y101,A^/*Y* ^S90,A^, and single copy in spores that inherit alleles from the same parent at both positions. Thus, to map the position of a transposed segment in one parental strain given knowledge of its position in the other, one can search for genes that show the requisite segregation pattern among spores in the GMS data for the known copy-number pattern in the aCGH data. This analysis was performed for TS15.1 with a custom Perl script (available upon request) that requires the user to input a gene that is close to, but not within, the transposed segment.

### Pulsed-Field Gel Electrophoresis and Southern Blotting

DNA was prepared for pulsed-field gel electrophoresis (PFGE) as previously described [66]. PFGE was performed for 38 hours at a gradient setting of 5.0 V/cm on a CHEF Mapper system (BioRad), with an initial and final switch time of 46.67 seconds and 2 minutes, 49.31 seconds, respectively. Chromosomes were visualized after ethidium bromide staining. The separated chromosomes were blotted onto a Hybond-XL membrane (GE Healthcare) and probed against a chromosome XV specific PCR fragment containing the YOL158C gene (SGD coordinates 15:19490-21310). The probe was labeled using Ready-to-Go DNA Labeling Beads (-dCTP) kit (GE Healthcare) and hybridized at 65°C for approximately 20 hours. The resulting signal was scanned and visualized using a Typhoon 9200 Variable Mode Imager (GE Healthcare).

### Paired-End Sequencing

Y101 and S90 genomic DNA were each run on a single lane of an Illumina GA2 sequencer for 36 cycles using the standard flow cell, yielding ∼28× coverage apiece. Here we report the results only for Y101. Raw Illumina GA2 image data was phased and filtered for quality using default GERALD parameters for unaligned reads (analysis: NONE, Use_Bases: 35). Sequencing reads were mapped back to the S288C reference sequence with ELAND. A custom script was written to identify clusters of paired-ends where one paired end mapped to a substantially different location than the other (available upon request). Both ends matched a unique string in the reference sequence for 61% of the reads in Y101. Putative TS were identified by at least an order of magnitude increase, relative to background, in the number of paired-ends within a region that mapped to a chromosomal location greater than 5 kb away. Two additional patterns were used to confirm putative TS. First, we required that there be paired ends spanning the predicted deletion. Second, we required that there be sets of paired-ends that map to the two different chromosomal locations on either side of both breakpoints (“reciprocity”).

### Comparison to Other *S. cerevisiae* Strains and Closely Related Species

Genome assembly and gene order data was obtained for *S. cerevisiae* strains RM11-1a [37] and YJM789 [32]. Gene order for the TS15.1 region in *S. paradoxus* and *S. bayanus* [40] was determined using the tiling data available from the Broad Institute (ftp://ftp-genome.wi.mit.edu/pub/annotation/fungi/comp_yeasts/S3b.Visualization_tiling/).

### Data Availability

Raw microarray data and images are publicly available from the UNC Microarray Database (UMD, https://genome.unc.edu), and microarray data and paired-end whole-genome sequencing data (for both Y101 and S90) are available through accession number GSE14223 at the NCBI Gene Expression Omnibus and Short Read Archive, respectively.

## Supporting Information

Table S1 Duplications and deletions relative to S288C. Probes adjacent or within 5 kb in the genome have been combined.
(0.17 MB DOC)

Table S2 PCR primers used to characterize TS15.1.
(0.08 MB DOC)

Table S3 Results of PCR assays to characterize TS15.1 in strains S288C, S90, and Y101, and all six tetrads.
(0.25 MB XLS)

Table S4 Genome rearrangements predicted by paired-end sequencing of Y101.
(0.03 MB XLS)
