# Supplementary material for: Systematic Identification of Balanced Transposition Polymorphisms in Saccharomyces cerevisiae
Source: PLoS Genet. 2009 Jun 5;5(6):e1000502. doi: 10.1371/journal.pgen.1000502 (PMC2682701; doi:10.1371/journal.pgen.1000502)
Supplement: Table S1 — Duplications and deletions relative to S288C. Probes adjacent or within 5 kb in the genome have been combined. (0.17 MB DOC) [file pgen.1000502.s001.doc]

**Table S1. Duplications and deletions relative to S288C.** Probes adjacent or within 5kb in the genome have been combined.

**1a. Deletions in Y101.** Previously identified deletions (from Lashkari et al. 1997) are highlighted.

| **Chromosome** | **ORF/Intergenic Boundaries**  **Left Right** | | **Coordinates in S288C**  **Left Right** | |
| --- | --- | --- | --- | --- |
| 1 | YAL064C-A | IYALWDELTA1 | 13364 | 23996 |
| 1 | YAR009C | YAR010C | 160597 | 165866 |
| 1 | YAR031W | IYAR033W | 186830 | 189426 |
| 1 | IYAR044W-1 | IYAR047C-1 | 197485 | 203403 |
| 1 | YAR053W | IYAR061W | 208357 | 218536 |
| 2 | YBL113C | IYBL111C | 280 | 5797 |
| 2 | IYBL101C | IYBLWTAU1 | 28171 | 36400 |
| 2 | YBR012C | YBR012W-A | 259104 | 261148 |
| 3 | YCL020W | YCL020W | 85101 | 86417 |
| 3 | IYCR104W | YCR107W | 308172 | 314974 |
| 4 | YDL246C | IYDL246C-1 | 8683 | 11650 |
| 4 | YDR035W | IYDR039C | 521810 | 535199 |
| 4 | IYDR170W-A-0 | IYDR170W-A-1 | 804516 | 806622 |
| 4 | IYDRWDELTA14 | IYDRCTY1-2 | 877785 | 884363 |
| 5 | IYEL073C-0 | IYEL073C-1 | 7560 | 10010 |
| 5 | YERWDELTA21 | IYERWDELTA1-2 | 444849 | 448987 |
| 7 | IYGL263W | IYGL262W | 3918 | 6298 |
| 7 | IYGLWDELTA7 | YGL051W | 402291 | 404392 |
| 7 | YGR038W | YDRCTY1-2C | 560683 | 566265 |
| 7 | YGR287C | IYGR291C | 1067226 | 1076583 |
| 8 | YHL050C | IYHL050C | 445 | 3715 |
| 8 | YHL008C | IYHL008C | 92622 | 95120 |
| 8 | YHR052W | YHR055C | 210840 | 214718 |
| 8 | IYHR211W | IYHR212C | 528614 | 539146 |
| 8 | IYHR214W-A | IYHRCDELTA16-A | 543488 | 550863 |
| 9 | IYIL083C | YILWTY3-1D | 204636 | 210292 |
| 9 | YIL015C-A | IYIL015C-A | 325210 | 325760 |
| 9 | YIR042C | IYIR042C | 435270 | 437049 |
| 10 | YJL219W | YJL216C | 19497 | 26086 |
| 10 | YJL115W | IYJLCDELTA3 | 195986 | 204419 |
| 10 | IYJR025C | YJR028W | 471368 | 479359 |
| 10 | IYJR152W | IYJR153W-0 | 720994 | 724532 |
| 12 | YLL067C | IYLL067C-1 | 585 | 6129 |
| 12 | IYLR035C-A-0 | IYLR035C-A-1 | 218907 | 220661 |
| 12 | IRDN5-2 | IYLR157C-B | 468930 | 475977 |
| 12 | IYLRCDELTA8 | IYLR162W-A | 481898 | 490899 |
| 12 | YLRWDELTA15 | IYLRWDELTA15 | 657449 | 658841 |
| 13 | YML039W | YML040W | 196628 | 197950 |
| 13 | YMR045C | IYMRCDELTA10 | 378606 | 378722 |
| 14 | IYNLCDELTA1-0 | IYNLCDELTA1-2 | 96925 | 100872 |
| 15 | YOL166C | YOL162W | 1000 | 10765 |
| 15 | IYOL140W | IYOL139C | 60015 | 61336 |
| 15 | TEL15R-5 | TEL15R-6 | 1088578 | 1091188 |
| 16 | YPLCDELTA10 | IYPLCTAU2 | 436241 | 444587 |
| 16 | IYPR194C | YPR204W | 926959 | 947696 |

**1b. Duplications in Y101.**

| **Chromosome** | **ORF/Integenic Boundaries**  **Left Right** | | **Coordinates in S288C**  **Left Right** | |
| --- | --- | --- | --- | --- |
| 1 | IYAR062W | YAR066W | 219135 | 221650 |
| 1 | YAR073W | YAR075W | 227732 | 229307 |
| 2 | YBL091C | IYBL091C | 47357 | 48834 |
| 2 | YBR284W | IYBR285W | 771195 | 774589 |
| 2 | YBR297W | IYBR298C | 800477 | 805309 |
| 3 | YCL034W | YCL032W | 61658 | 64481 |
| 3 | YCL008C | SNR43 | 105959 | 107708 |
| 3 | YCR020W-B | YCR021C | 155332 | 157099 |
| 3 | IYCR095C-1 | YCRWOMEGA3 | 290312 | 292171 |
| 3 | IYCR101C | YCR103C | 303023 | 306310 |
| 4 | YDR176W | IYDR177W | 814445 | 817936 |
| 4 | IYDR431W | YDR433W | 1328681 | 1330028 |
| 4 | IYDR474C | YDR476C | 1409141 | 1411113 |
| 4 | IYDR542W | YDR544C | 1523607 | 1525502 |
| 6 | IYFL062W-0 | IYFL054C | 7589 | 23423 |
| 7 | YGR224W | YGR225W | 942806 | 946926 |
| 8 | IYHR086W | YHR088W | 279710 | 282383 |
| 8 | YHRCDELTA8 | IYHRWDELTA9 | 358665 | 359701 |
| 8 | YHR137W | YHR139C | 375710 | 379200 |
| 8 | IYHRCDELTA10 | YHR146W | 389503 | 391698 |
| 8 | IYHR163W | IYHR164C | 424467 | 429700 |
| 8 | IYHR213W-B | IYHR214W | 540694 | 543003 |
| 8 | YHR215W | YHR217C | 552095 | 557038 |
| 10 | YJL214W | YJL214W | 26887 | 28596 |
| 10 | YJR154W | YJR156C | 725478 | 729290 |
| 11 | YKL160W | IYKL159C | 153270 | 154988 |
| 11 | IYKL042W | YKL040C | 359211 | 361468 |
| 12 | YLR460C | IYLR460C-0 | 1059753 | 1061909 |
| 13 | YMR262W | IYMR263W | 793725 | 795813 |
| 14 | IYNL336W-0 | IYNL333W | 9499 | 14831 |
| 14 | YNL016W | IYNL016W | 602903 | 605395 |
| 16 | YPL279C | YPL273W | 13228 | 26064 |

**1c. Deletions in S90.**

| **Chromosome** | **ORF/Intergenic Boundaries**  **Left Right** | | **Coordinates in S288C**  **Left Right** | |
| --- | --- | --- | --- | --- |
| 4 | YDRWDELTA10 | YDR107C | 668574 | 671027 |
| 7 | IYGRCDELTA18 | IYGRDELTA18 | 574686 | 574884 |
| 12 | YLR154C | IYLRWDELTA6-0 | 4447983 | 450403 |
| 12 | IYLR272C | IYLRWDELTA17 | 687191 | 689083 |
| 13 | IYMR140W | IYMR141C | 549174 | 550158 |
| 14 | YNL001W | YNR001C | 627453 | 631058 |
| 15 | IYOR007C | IYOLCDELTA9 | 339967 | 341234 |
| 16 | YPLCTY4-1E | IYPLCTAU2 | 441868 | 444587 |

**1d. Duplications in S90**

| **Chromosome** | **ORF/Intergenic Boundaries**  **Left Right** | | **Coordinates in S288C**  **Left Right** | |
| --- | --- | --- | --- | --- |
| 2 | YBL113C | IYBL109W-0 | 280 | 7144 |
| 2 | YBR261C | YBR263W | 734789 | 737693 |
| 3 | YCL034W | YCL032W | 61658 | 64481 |
| 3 | YCR073W-A | YCR075C | 246970 | 248808 |
| 4 | YDR432W | YDR433W | 1328771 | 1330028 |
| 4 | IYDR542W | IYDR544C | 1523607 | 1526329 |
| 5 | IYEL077C | IYEL074W | 4095 | 7226 |
| 5 | YEL068C | YEL065W | 25646 | 29543 |
| 6 | YFL068W | IYFL063W | 53 | 6416 |
| 7 | YGL236C | YGL234W | 53756 | 58889 |
| 7 | IYGR067C | IYGR067C | 624765 | 625326 |
| 8 | INTYHL050C | IYHL049C-1 | 1893 | 6390 |
| 8 | IYHR212C | IYHR214W | 538089 | 543003 |
| 8 | YHR216W | IYHR219W | 554392 | 562418 |
| 9 | TEL9L | IYIL177C-0 | 95 | 7473 |
| 10 | YJL225C | IYJL225C-0 | 466 | 7456 |
| 12 | YLL067C | IYLL066C-1 | 585 | 11728 |
| 12 | RDN37-1 | RDN37-1 | 451780 | 458433 |
| 12 | RDN25-1A | IRDN371 | 451785 | 459677 |
| 12 | YLR437C | YLR439W | 1011617 | 1015445 |
| 12 | IYLR461W-1 | YLR465C | 1064602 | 1067349 |
| 13 | YML133C | IYML133C-1 | 461 | 7234 |
| 14 | IYNL339C | IYNL337W | 6083 | 8320 |
| 15 | YOL087C | YOL087C | 155286 | 158636 |
| 15 | TEL15R-1 | TEL15R-5 | 1083317 | 1089862 |
| 16 | IYPR201W-1 | YPR204W | 942084 | 947696 |

1. Lashkari DA, DeRisi JL, McCusker JH, Namath AF, Gentile C, et al. (1997) Yeast microarrays for genome wide parallel genetic and gene expression analysis. Proc Natl Acad Sci U S A 94: 13057-13062.
