# Supplementary material for: Systematic Identification of Balanced Transposition Polymorphisms in Saccharomyces cerevisiae
Source: PLoS Genet. 2009 Jun 5;5(6):e1000502. doi: 10.1371/journal.pgen.1000502 (PMC2682701; doi:10.1371/journal.pgen.1000502)
Supplement: Table S2 — PCR primers used to characterize TS15.1. (0.08 MB DOC) [file pgen.1000502.s002.doc]

**Table S2. PCR primers used to characterize TS-15.1**.

2a. Primers originally designed to amplify probes for the microarray.

| No. | Probe | Forward Primer | Reverse Primer |
| --- | --- | --- | --- |
| 1 | IYOL166C | TGCACATACGCCGTTAAGTAG | ATAATTTTTCAGTAATCAACTACGCA |
| 2 | YOL165C | GCTAGGCATTTCGGTATGGC | TTATTCGTACCATATTTTTG |
| 3 | IYOL165C | TAGCCATTGGAATAATATCACGCT | CACCCAATTCTCACCATCAAAG |
| 4 | IYOLCDELTA1 | TTTCGTTCCAACATCAATACCTCT | CGTAAACGTCCAGTTAATCTTCCA |
| 5 | YOLWTAU1 | AAAGATATCTGTCCCTTCATTAAGAC | GCACATTCTCTTATTGTTGATAATTG |
| 6 | IYOLWTAU1-0 | AAGAGAATGTGCTACCCTGAGTGA | CACACATCCTGCTACCAGTCG |
| 7 | IYOLWTAU1-1 | TGTGTGAATGCTCCTCTGACG | GGCACCAATCATATCCGGTT |
| 8 | YOL164W | TTGGTGCCTTTAAGAGGAAC | AAGTGCTTCCCCCCATAGGC |
| 9 | IYOL164W | ATCAGAAATCGGCGAGAAACTT | CATTTGCAGAGGAATCCAACTATC |
| 10 | Yol163w | TGGTCCCTAGTGGCTACGTT | TGTTATCCCCTGCCATAACATT |
| 11 | Yol162w | ACGTTTTGGCCACATACCTG | CGAGTCTACGTGAACCAGCA |
| 12 | IYOL162W | GGATACAATCTCCATGTTATGTATTT | CCATAGACAGTGAAAAGTAATGAAAAAT |
| 13 | YOL161C | CAAATTAACTTCAATCGCTG | TTGCGATAGTGTAGATACCA |
| 14 | IYOL161C-0 | TTGTTTTGTTTTTTAGTGCTGATATGA | AGAATTAAGAATACGTTTACCATGGAAC |
| 15 | IYOL161C-1 | GCTTTAAAAAACAGGCACATATGTT | AAACTGTCATATAGAATCGTGCAAGA |
| 16 | YOL160W | ATATAGCATTCATTTGTCTT | AAGAAGAGCAATAATCTTGG |
| 17 | IYOL160W-0 | GAATAAGCCATCACATAGATGCG | GAACGAGTGTAAAAACAGCAAGGT |
| 18 | IYOL160W-1 | CGTTCCAAGTAAAAAATGCTCATC | GATACTGGAACCCTTTTATGACACAT |
| 20 | IYOL159C | AAGGGAACCATTTTTTACTAAGCCT | TTCACCAAATTCTCTATTTCAACAGT |
| 21 | YOLCDELTA2 | TGGTGAATAATTGGATAATTGTTAGG | CGACATAAAGATAGAGAAACAGCCA |
| 23 | YOL158C | TGGAAACTGATCACTCTAGG | TATCCAATTACACGACGGAG |
| 24 | IYOL158C | TTCAGAGAGACATTCACGAGCAC | AATGATTGATGATTGTGACGACAA |
| 25 | YOL157C | TATTTCTTCTGCACATCCAG | CAGATATGTAAATTCTGCCC |
| 26 | iyol157c | ATATGTCTTTTACGTGCCTA | ATTGAGTAATAACAAAAGCG |
| 27 | Yol156w | TGAGTTATCTACTACCATGTC | ACCCTAAGAGGAAACGTCTCTGA |
| 28 | iyol156w-0 | ATGATATTTTTTGAAGCCTTGAAAAATA | ACGCATAATTGAGTTTTAACTTTCG |
| 29 | iyol156w-1 | GATATCTTTTCCTGCGGGTTCTC | TGCGTTTTATATACGCTTTGTGTAA |
| 31 | IYOL155C-0 | TCTCTTTGACAGGCGAGATCTTAC | AGGCCAACCATAAACACCTCA |
| 32 | IYOL155C-1 | ACGGTCCGATATGTGTAGTGGTT | GTTTAGTAGTTGTGTGTGGATTTGGTT |
| 33 | YOL154W | GTTCTCTTCCGGCAAATCTA | AAGTTACCTAGACAGCCACC |
| 34 | IYOL154W | AACCACATCATTACTTCCACAGAA | CATGATTAATGGGCGAATTTTTT |
| 35 | YOL153C | ACAGAGACTCATCACGCTCC | CATGGCCATACTCGTTGACA |
| 36 | IYOL153C-1 | TAACATCAGCCCATCACCCA | TTTGAAATATCCCTTTACTTGCAATAA |
| 37 | YOL152W | AAGAAAGAGATTTGGTTTTA | ATGGTGGATGCCATCTGTTT |
| 38 | IYOL152W | ATTCAAGCATTTCGGCAAAAC | TTACGGGCGTGTGATACTGC |
| 39 | YOL151W | AGTTTTCGTTTCAGGTGCTA | TGCCCTCAAATTTTAAAATT |

| No. | Probe | Forward Primer | Start | Reverse Primer | Start | Internal Primer | Start |
| --- | --- | --- | --- | --- | --- | --- | --- |
| 14 | iyol161c-0 | ATGTTTCATCTTTCGCACCATCTTC | 12803 | CATGGAACGGTGACTTACAACGAAT | 13071 | TTCTGCCACTAAGAAATGGTTACAGTT | 12571 |
| 19 | yol159c | GGAGTTGGATGAGCGCAAGC | 17350 | TGGTTCCCTTATTTGGTTTATTTTGC | 17769 | CGGCGATCAGCAATCTTTGA | 17567 |
| 22 | iyolcdelta2 | GAAAACGACACTGCAAAGAGTGGAT | 18605 | TCAATGAGATTATTCGCACCTGTCA | 19207 | GCAGTAACCCCACGAAATTTTAACC | 18939 |
| 30 | yol155c | TCAAACCTGCGGCAATACCC | 28758 | TCCACTTCATTCGGTGCTTCC | 29620 | GAGGCCCCTGAAGCCACAA | 29172 |
| 30 | yol155c | CGCTGGAAGCAACAACACCTC | 29733 | TGCAACTGCAACTGCTTCCAA | 30635 | CCGTCTCTGGTTTGCAGCGTA | 30174 |
| 30 | yol155c | AGGCAGTGGAAGCCGATGAG | 30708 | TTCAAGCCGCTTTGGCTTTG | 31564 | ATCCGCCTCCGGTTCATCTT | 3094 |
| 31 | iyol155c-0 | GCGGACTCCGAATACGCAAC | 31765 | TCGGCAATCAATGACGATGC | 3306 | GCTTCAATGGCCTGCTCGAT | 32492 |

**2b. Newly designed primer combinations**.
